# Supplementary material for: Drug repurposing for aging research using model organisms
Source: Aging Cell. 2017 Jun 16;16(5):1006–15. doi: 10.1111/acel.12626 (PMC5595691; doi:10.1111/acel.12626)
Supplement: Supplementary file 7 — Data S1 Zip‐Archive of all report cards. [file ACEL-16-1006-s007.zip › RC_FAD.pdf]

FAD

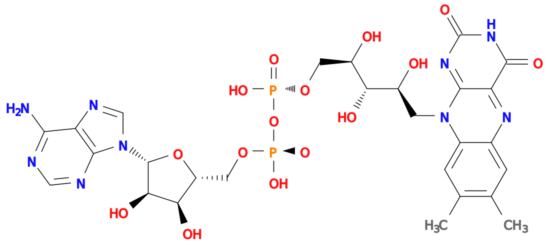

Database identifiers

ChEMBLCompound	CHEMBL1232653
DrugBank	DB03147
CHEBI	16238

Ranking

	Rank	Score
Drosophila	266/697	0.621
C. elegans	577/591	0.0

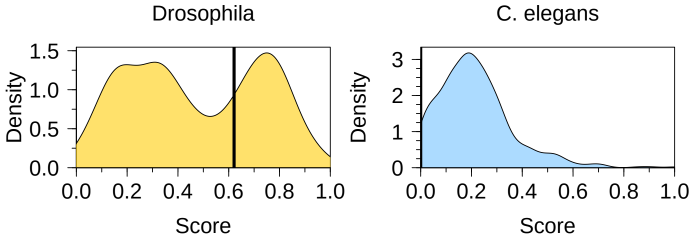

	Ageing implication	Domain conservation	Binding site conservation	Binding affinity	Bioavailability	Lipinski	Promiscuity	Purchasability	Drug approval	**Total**
Drosophila	1.0	0.956	0.973	0.951	(0.9)	-0.15	-0.1	0.0	0.075	0.621
C. elegans	1.0	0.958	0.943	0.951	0.203	-0.15	-0.1	0.0	0.075	0.0

Names

- **FAD**
- Adenosine 5'-(trihydrogen pyrophosphate), 5'-5'-ester with riboflavine
- adenosine 5'-3-(riboflavin-5'-yl) dihydrogen diphosphate
- Flavin adenine dinucleotide
- Riboflavin 5'-(trihydrogen diphosphate), 5'-5'-ester with adenosine
- Riboflavin 5'-adenosine diphosphate

Roles

human metabolite

Status

Approved drug (according to ChEMBL)	No
Classification (according to DrugBank)	approved
Administration Route	Ophthalmic
Number of Rule of 5 violations	3
Binding affinity to original target in log units (RF-Score prediction)	7.97
Burns *C. elegans* bioavailability prediction	-8.32

Medical Information

Indicatation: Used to treat eye diseases caused by vitamin B2 deficiency, such as keratitis and blepharitis.
Toxicity: Oral mouse LD50 > 7000 mg/kg.

(Information from DrugBank)

Compound Target Characteristics

Apoptosis-inducing factor 1, mitochondrial

Best gene implication in ageing for this target family came from gene O95831 via mapping the annotation from Ensembl ENSG00000156709 via mapping the annotation from EntrezGene 9131 via mapping the annotation from GenAgeHuman 0135 annotated in GenAge release 17.

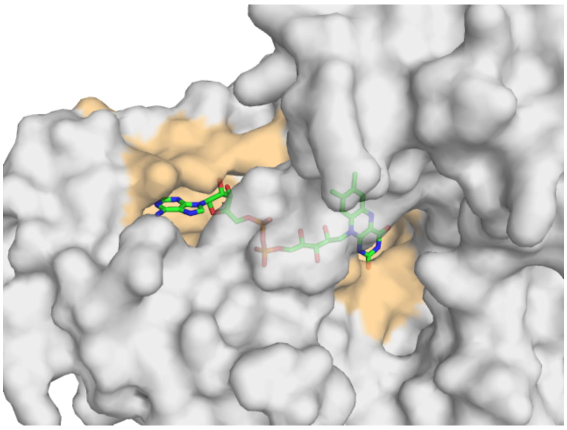

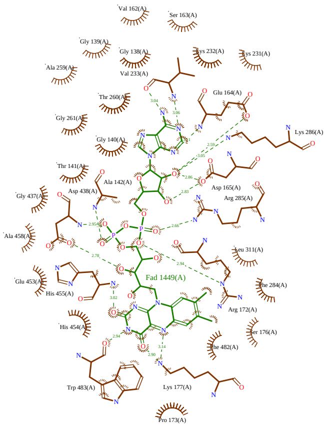

protein	amino acids contacts (binding site)
PDB:1m6i:chainA:O95831	I G G G T A V S E D R P S K K K V A T G F R K L E N G D E H H A F W
PDB:4bur:chainA:O95831	I G G G T A V S E D R P S K K K V A T G F R K L E N G D E H H A F W
PDB:4fdc:chainB:O95831	I G G G T A V S E D R P S K K K V A T G F R K L E N G D E H H A F W
PDB:4lii:chainA:O95831	I G G G T A V S E D R P S K K K V A T G F R K L E N G D E H H A F W
sp:O95831:AIFM1_HUMAN	I G G G T A V S E D R P S K K K V A T G F R K L E N G D E H H A F W
sp:Q9JM53:AIFM1_RAT	I G G G T A V S E D R P S K K K V A T G F R K L E N G D E H H A F W
tr:B1AU25:B1AU25_MOUSE	I G G G T A V S E D R P S K K K V A T G F R K L E N G D E H H A F W
sp:Q9Z0X1:AIFM1_MOUSE	I G G G T A V S E D R P S K K K V A T G F R K L E N G D E H H A F W
sp:Q9VQ79:AIFM1_DROME	I G G G T A I S N E R P S K F S V A T G Y R T I E N G D E H H S F W
tr:E8NH83:E8NH83_DROME	I G G G T A I S N E R P S K F S V A T G Y R T I E N G D E H H S F W
tr:K8ESD4:K8ESD4_CAEEL	I G S G T A I G E E R P S K R K A A T G F H Y L E N G A S S W A F F
tr:Q8HJ11:Q8HJ11_CAEEL	I G S G T A I G E E R P S K R K A A T G F H Y L E N G A S S W A F F
tr:Q9U229:Q9U229_CAEEL	I G S G T A I G E E R P S K R K A A T G F H Y L E N G A S S W A F F

protein	whole protein ident	simil	domain-based ident	simil	contact-based ident	simil
PDB:1m6i:chainA:O95831	1.0	1.0	1.0	1.0	1.0	1.0
PDB:4bur:chainA:O95831	1.0	1.0	1.0	1.0	1.0	1.0
PDB:4fdc:chainB:O95831	1.0	1.0	1.0	1.0	1.0	1.0
PDB:4lii:chainA:O95831	1.0	1.0	1.0	1.0	1.0	1.0
sp:O95831:AIFM1_HUMAN	1.0	1.0	1.0	1.0	1.0	1.0
sp:Q9JM53:AIFM1_RAT	0.93	0.98	0.96	0.99	1.0	1.0
tr:B1AU25:B1AU25_MOUSE	0.88	0.95	0.95	0.99	1.0	1.0
sp:Q9Z0X1:AIFM1_MOUSE	0.92	0.98	0.95	0.99	1.0	1.0
sp:Q9VQ79:AIFM1_DROME	0.37	0.66	0.51	0.82	0.74	0.85
tr:E8NH83:E8NH83_DROME	0.36	0.64	0.51	0.82	0.74	0.85
tr:K8ESD4:K8ESD4_CAEEL	0.29	0.58	0.37	0.71	0.62	0.77
tr:Q8HJ11:Q8HJ11_CAEEL	0.25	0.55	0.36	0.7	0.62	0.77
tr:Q9U229:Q9U229_CAEEL	0.25	0.55	0.37	0.71	0.62	0.77

AIF (FBgn0031392) associated phenotypes
decreased cell death, dominant, immune response defective, increased cell death, partially, small body

(Information from FlyBase)

AIF (UniProt:Q9VQ79) annotation
Function: Probable NADH oxidoreductase (By similarity). Mitochondrial effector of cell death that plays roles in developmentally regulated cell death and normal mitochondrial function. (, PubMed:18309327).
Cofactor: FAD
Subcellular location: Mitochondrion intermembrane space (PubMed:18309327).
Developmental stage: Expressed both maternally and zygotically throughout development with slightly lower levels during pupation. (PubMed:18309327).
Disruption phenotype: Loss of zygotic expression results in decreased embryonic cell death and the persistence of differentiated neuronal cells along the ventral nerve cord at late embryonic stages. Embryos that do hatch undergo growth arrest at early larval stages, accompanied by mitochondrial respiratory dysfunction. (PubMed:18309327).

(Information from UniProt)

Glutathione reductase, mitochondrial

Best gene implication in ageing for this target family came from gene P91938 annotated in UniProt release 2014_02. Annotation GO 8340 (determination of adult lifespan) was Inferred from Mutant Phenotype

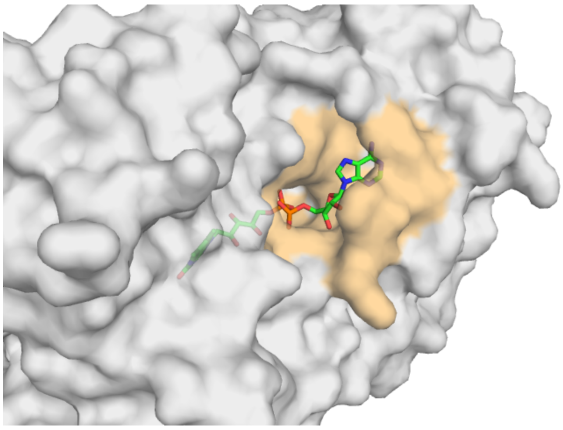

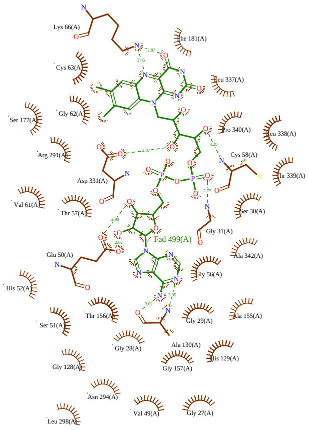

protein	amino acids contacts (binding site)
PDB:1bwc:chainA:P00390	I G G G S G V E S H K G T C V G C K G H A A T G G S F Y I R N L V G D L L T P
PDB:1dnc:chainA:P00390	I G G G S G V E S H K G T C V G X K G H A A T G G S F Y I R N L V G D L L T P
PDB:1gra:chainA:P00390	I G G G S G V E S H K G T C V G C K G H A A T G G S F Y I R N L V G D L L T P
PDB:1grb:chainA:P00390	I G G G S G V E S H K G T C V G C K G H A A T G G S F Y I R N L V G D L L T P
PDB:1gre:chainA:P00390	I G G G S G V E S H K G T C V G C K G H A A T G G S F Y I R N L V G D L L T P
PDB:1grf:chainA:P00390	I G G G S G V E S H K G T C V G C K G H A A T G G S F Y I R N L V G D L L T P
PDB:1grg:chainA:P00390	I G G G S G V E S H K G T X V G C K G H A A T G G S F Y I R N L V G D L L T P
PDB:1grt:chainA:P00390	I G G G S G V E S H K G T C V G C K G H A A T G G S F Y I R N L V G D L L T P
PDB:1gsn:chainA:P00390	I G G G S G V E S H K G T C V G X K G H A A T G G S F Y I R N L V G D L L T P
PDB:1xan:chainA:P00390	I G G G S G V E S H K G T C V G C K G H A A T G G S F Y I R N L V G D L L T P
PDB:2aaq:chainA:P00390	I G G G S G V E S H K G T C V G C K G H A A T G G S F Y I R N L V G D L L T P
PDB:2gh5:chainA:P00390	I G G G S G V E S H K G T C V G C K G H A A T G G S F Y I R N L V G D L L T P
PDB:2grt:chainA:P00390	I G G G S G V E S H K G T C V G C K G H A A T G G S F Y I R N L V G D L L T P
PDB:3grs:chainA:P00390	I G G G S G V E S H K G T C V G C K G H A A T G G S F Y I R N L V G D L L T P
PDB:3grt:chainA:P00390	I G G G S G V E S H K G T C V G C K G H A A T G G S F Y I R N L V G D L L T P
PDB:4gr1:chainA:P00390	I G G G S G V E S H K G T C V G C K G H A A T G G S F Y I R N L V G D L L T P
PDB:4grt:chainA:P00390	I G G G S G V E S H K G T C V G C K G H A A T G G S F Y I R N L V G D L L T P
PDB:5grt:chainA:P00390	I G G G S G V E S H K G T C V G C K G H A A T G G S F Y I R N L V G D L L T P
sp:P00390:GSHR_HUMAN	I G G G S G V E S H K G T C V G C K G H A A T G G S F Y I R N L V G D L L T P
tr:F1LRE1:F1LRE1_RAT	- - - - - - - - - - - - - - - - - - G Y A A T G G S F Y I R N L V G D L L T P
sp:P47791:GSHR_MOUSE	I G G G S G V E S H K G T C V G C K G Y A A T G G S F Y I R N L V G D L L T P
sp:P91938:TRXR1_DROME	I G G G S A L D F V K G T C V G C K G L G A V G G S F Y I R L L V G D E L T P
tr:Q86DB5:Q86DB5_CAEEL	I G G G S G I E S G R G T C V G C K G R A A V G G S F Y I R L L V G D L L T P
tr:Q93379:Q93379_CAEEL	I G G G S G I E S G R G T C V G C K G R A A V G G S F Y I R L L V G D L L T P
sp:P41921:GSHR_YEAST	I G G G S G V E A K A G T C V G C K G W A A T G G S F Y I R H M L G D E L T P

	whole protein		domain-based		contact-based	
protein	ident	simil	ident	simil	ident	simil
PDB:1bwc:chainA:P00390	1.0	1.0	1.0	1.0	1.0	1.0
PDB:1dnc:chainA:P00390	1.0	1.0	1.0	1.0	0.98	0.96
PDB:1gra:chainA:P00390	1.0	1.0	1.0	1.0	1.0	1.0
PDB:1grb:chainA:P00390	1.0	1.0	1.0	1.0	1.0	1.0
PDB:1gre:chainA:P00390	1.0	1.0	1.0	1.0	1.0	1.0
PDB:1grf:chainA:P00390	1.0	1.0	1.0	1.0	1.0	1.0
PDB:1grg:chainA:P00390	1.0	1.0	1.0	1.0	0.98	0.96
PDB:1grt:chainA:P00390	1.0	1.0	1.0	1.0	1.0	1.0
PDB:1gsn:chainA:P00390	0.99	0.99	0.99	0.99	0.98	0.96
PDB:1xan:chainA:P00390	1.0	1.0	1.0	1.0	1.0	1.0
PDB:2aaq:chainA:P00390	1.0	1.0	1.0	1.0	1.0	1.0
PDB:2gh5:chainA:P00390	1.0	1.0	1.0	1.0	1.0	1.0
PDB:2grt:chainA:P00390	1.0	1.0	1.0	1.0	1.0	1.0
PDB:3grs:chainA:P00390	1.0	1.0	1.0	1.0	1.0	1.0
PDB:3grt:chainA:P00390	1.0	1.0	1.0	1.0	1.0	1.0
PDB:4gr1:chainA:P00390	1.0	1.0	1.0	1.0	1.0	1.0
PDB:4grt:chainA:P00390	1.0	1.0	1.0	1.0	1.0	1.0
PDB:5grt:chainA:P00390	1.0	1.0	1.0	1.0	1.0	1.0
sp:P00390:GSHR_HUMAN	1.0	1.0	1.0	1.0	1.0	1.0
tr:F1LRE1:F1LRE1_RAT	0.68	0.76	0.78	0.86	0.56	0.12
sp:P47791:GSHR_MOUSE	0.8	0.89	0.89	0.97	0.98	0.98
sp:P91938:TRXR1_DROME	0.28	0.64	0.36	0.75	0.77	0.8
tr:Q86DB5:Q86DB5_CAEEL	0.51	0.74	0.6	0.86	0.86	0.91
tr:Q93379:Q93379_CAEEL	0.5	0.73	0.6	0.86	0.86	0.91
sp:P41921:GSHR_YEAST	0.44	0.73	0.51	0.81	0.81	0.87

Trxr-1 (FBgn0020653) associated phenotypes

aging defective, developmental rate defective, lethal - all die before end of P-stage, lethal - all die before end of pupal stage, neuroanatomy defective, partially, partially lethal - majority die, some die during pharate adult stage, some die during pupal stage, stress response defective

(Information from FlyBase)

Trxr-1 (UniProt:P91938) annotation

Function: Thioredoxin system is a major player in glutathione metabolism, due to the demonstrated absence of a glutathione reductase. Functionally interacts with the Sod/Cat reactive oxidation species (ROS) defense system and thereby has a role in preadult development and life span. Lack of a glutathione reductase suggests antioxidant defense in Drosophila, and probably in related insects, differs fundamentally from that in other organisms. (PubMed:11158675, PubMed:11525742, PubMed:11796729).

Cofactor: FADNote=Binds 1 FAD per subunit.;

Biophysicochemical properties: Kinetic parameters: KM=6.5 uM for NADPH (at pH 7.4) (PubMed:11158675, PubMed:11782468, PubMed:11796729, PubMed:11877442, PubMed:17385893, PubMed:18211101, PubMed:18991392); KM=1 uM for NADPH (at pH 7.4, 2 mM EDTA, 100 mM KPO(4)) (PubMed:11158675, PubMed:11782468, PubMed:11796729, PubMed:11877442, PubMed:17385893, PubMed:18211101, PubMed:18991392); KM=1 uM for NADPH (isoform B at pH 7.4, 2 mM EDTA, 100 mM KPO(4)) (PubMed:11158675, PubMed:11782468, PubMed:11796729, PubMed:11877442, PubMed:17385893, PubMed:18211101, PubMed:18991392); KM=7.0 uM for dhd (at pH 7.4, 200 uM NADPH, 100 mM KPO(4)) (PubMed:11158675, PubMed:11782468, PubMed:11796729, PubMed:11877442, PubMed:17385893, PubMed:18211101, PubMed:18991392); KM=141 uM for dhd (at pH 7.0, 0.15 mM NADPH, 1 mM EDTA, 1 mg/ml insulin, 50 mM KPO(4), 25 degrees Celsius) (PubMed:11158675, PubMed:11782468, PubMed:11796729, PubMed:11877442, PubMed:17385893, PubMed:18211101, PubMed:18991392); KM=7 uM for dhd (at pH 7.4, 2 mM EDTA, 100 mM KPO(4)) (PubMed:11158675, PubMed:11782468, PubMed:11796729, PubMed:11877442, PubMed:17385893, PubMed:18211101, PubMed:18991392); KM=19 uM for dhd (isoform B at pH 7.4, 2 mM EDTA, 100 mM KPO(4)) (PubMed:11158675, PubMed:11782468, PubMed:11796729, PubMed:11877442, PubMed:17385893, PubMed:18211101, PubMed:18991392); KM=5.9 uM for Trx-2 (at pH 7.4, 100 uM NADPH, 2 mM EDTA, 100 mM KPO(4)) (PubMed:11158675, PubMed:11782468, PubMed:11796729, PubMed:11877442, PubMed:17385893, PubMed:18211101, PubMed:18991392); KM=310 uM for 5,5'-dithiobis-2-nitrobenzoic acid (DTNB) (at pH 7.4, 100 uM NADPH, 100 mM KPO(4)) (PubMed:11158675, PubMed:11782468, PubMed:11796729, PubMed:11877442, PubMed:17385893, PubMed:18211101, PubMed:18991392); KM=0.17 mM for DTNB (at pH 7.0, 0.2 mM NADPH, 10 mM EDTA, 100 mM KPO(4), 25 degrees Celsius) (PubMed:11158675, PubMed:11782468, PubMed:11796729, PubMed:11877442, PubMed:17385893, PubMed:18211101, PubMed:18991392); KM=380 uM for DTNB (at pH 7.4, 2 mM EDTA, 100 mM KPO(4)) (PubMed:11158675, PubMed:11782468, PubMed:11796729, PubMed:11877442, PubMed:17385893, PubMed:18211101, PubMed:18991392); KM=410 uM for DTNB (isoform B at pH 7.4, 2 mM EDTA, 100 mM KPO(4)) (PubMed:11158675, PubMed:11782468, PubMed:11796729, PubMed:11877442, PubMed:17385893, PubMed:18211101, PubMed:18991392); KM=675 uM for methylseleninate (100 uM NADPH) (PubMed:11158675, PubMed:11782468, PubMed:11796729, PubMed:11877442, PubMed:17385893, PubMed:18211101, PubMed:18991392); Vmax=24.3 umol/min/mg enzyme toward NADPH (at pH 7.4) (PubMed:11158675, PubMed:11782468, PubMed:11796729, PubMed:11877442, PubMed:17385893, PubMed:18211101, PubMed:18991392); Vmax=16 umol/min/mg enzyme toward Trx-2 (at pH 7.4, 100 uM NADPH, 2 mM EDTA, 100 mM KPO(4), 25 degrees Celsius) (PubMed:11158675, PubMed:11782468, PubMed:11796729, PubMed:11877442, PubMed:17385893, PubMed:18211101, PubMed:18991392); Note=Measurements were conducted with isoform A unless noted otherwise.; pH dependence: Optimum pH is 7.6 for isoform A with Trx-2 and NADPH as substrates. (PubMed:11158675, PubMed:11782468, PubMed:11796729, PubMed:11877442, PubMed:17385893, PubMed:18211101, PubMed:18991392);

Subunit: Homodimer. (PubMed:17385893).

Subcellular location: Isoform B: Mitochondrion.

Subcellular location: Isoform A: Cytoplasm.

Tissue specificity: During embryogenesis, expression is seen in germ cell progenitors, developing midgut, hindgut and proventriculus. (PubMed:11525742).

Developmental stage: Expressed both maternally and zygotically during all stages of development, highest expression during adult stages. (PubMed:11525742, PubMed:11796729).

Miscellaneous: The active site is a redox-active disulfide bond.
Caution: Was originally thought to be a glutathione reductase. (PubMed:9056265).

(Information from UniProt)

gsr-1 (WBGene00008117) associated phenotypes

lethal, sterile

(Information from WormBase)

Dihydrolipoyl dehydrogenase, mitochondrial

Best gene implication in ageing for this target family came from gene O17953 via mapping the annotation from WormBase WBGene00010794 annotated in WormBase 2014-03-11. Annotation GO 8340 (determination of adult lifespan) was Inferred from Mutant Phenotype

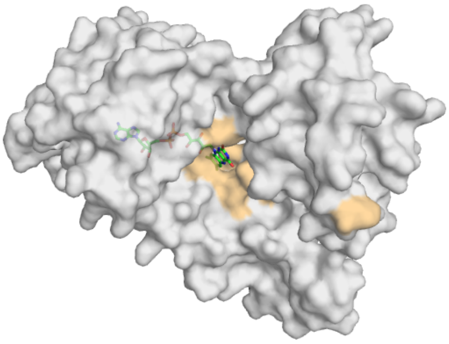

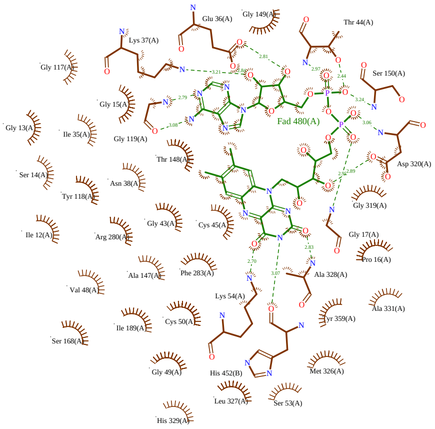

protein	amino acids contacts (binding site)
PDB:1zmc:chainA:P09622	I G S G P G G I E K N G T C V G C S K G Y G A T G S S I R F G D M L A H A Y H
PDB:1zmd:chainA:P09622	I G S G P G G I E K N G T C V G C S K G Y G A T G S S I R F G D M L A H A Y H
PDB:2f5z:chainA:P09622	I G S G P G G I E K N G T C V G C S K G Y G A T G S S I R F G D M L A H A Y H
sp:P09622:DLDH_HUMAN	I G S G P G G I E K N G T C V G C S K G Y G A T G S S I R F G D M L A H A Y H
sp:Q6P6R2:DLDH_RAT	I G S G P G G I E K N G T C V G C S K G F G A T G S S I R F G D M L A H A Y H
sp:O08749:DLDH_MOUSE	I G S G P G G I E K N G T C V G C S K G F G A T G S S I R F G D M L A H A Y H
tr:Q9VVL7:Q9VVL7_DROME	I G S G P G G V E K E G T C V G C S K G F G A T G S S I R Y G D M L A H A Y H
sp:O17953:DLDH_CAEEL	I G G G P G G V E K N G T C V G C S K G F A A S G S S I R Y G D M L A H A Y H
sp:P09624:DLDH_YEAST	I G G G P A G V E K R G T C V G C S K G N G A T G S S I R Y G D M L A H A Y H

	whole protein		domain-based		contact-based	
protein	ident	simil	ident	simil	ident	simil
PDB:1zmc:chainA:P09622	1.0	1.0	1.0	1.0	1.0	1.0
PDB:1zmd:chainA:P09622	1.0	1.0	1.0	1.0	1.0	1.0
PDB:2f5z:chainA:P09622	1.0	1.0	1.0	1.0	1.0	1.0
sp:P09622:DLDH_HUMAN	1.0	1.0	1.0	1.0	1.0	1.0
sp:Q6P6R2:DLDH_RAT	0.95	0.99	0.95	0.99	0.97	0.99
sp:O08749:DLDH_MOUSE	0.95	0.99	0.95	0.99	0.97	0.99
tr:Q9VVL7:Q9VVL7_DROME	0.65	0.86	0.7	0.91	0.9	0.97
sp:O17953:DLDH_CAEEL	0.68	0.88	0.72	0.91	0.85	0.94
sp:P09624:DLDH_YEAST	0.53	0.82	0.57	0.85	0.85	0.91

CG7430 (UniProt:Q9VVL7) annotation

Cofactor: FAD Evidence=(RuleBase:RU003692); Note=Binds 1 FAD per subunit. (RuleBase:RU003692);
Miscellaneous: The active site is a redox-active disulfide bond. (RuleBase:RU003692).

(Information from UniProt)

dld-1 (WBGene00010794) associated phenotypes
lethal, sterile

(Information from WormBase)

dld-1 (UniProt:O17953) annotation
Cofactor: FADNote=Binds 1 FAD per subunit. ;
Subcellular location: Mitochondrion matrix
Miscellaneous: The active site is a redox-active disulfide bond.

(Information from UniProt)

Dihydrolipoyl dehydrogenase, mitochondrial

Best gene implication in ageing for this target family came from gene O17953 via mapping the annotation from WormBase WBGene00010794 annotated in WormBase 2014-03-11. Annotation GO 8340 (determination of adult lifespan) was Inferred from Mutant Phenotype

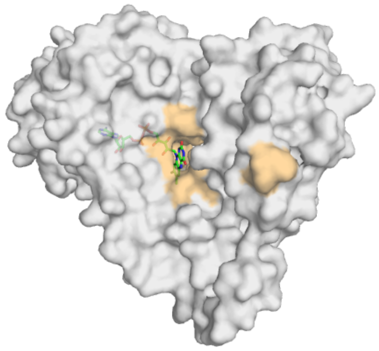

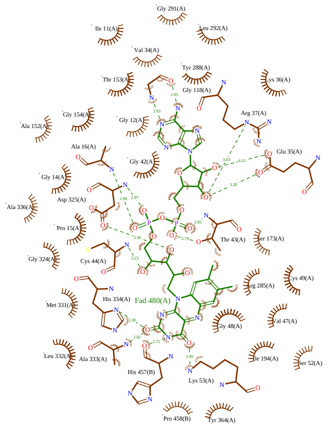

protein	amino acids contacts (binding site)
PDB:1jeh:chainA:P09624	I G G P A G V E K R G G T C V G C S K G N G A T G S S I R Y A G L G D M L A H
PDB:1v59:chainA:P09624	I G G P A G V E K R G G T C V G C S K G N G A T G S S I R Y A G L G D M L A H
sp:P09622:DLDH_HUMAN	I G G P G G I E K N G G T C V G C S K G Y G A T G S S I R F K N L G D M L A H
sp:Q6P6R2:DLDH_RAT	I G G P G G I E K N G G T C V G C S K G F G A T G S S I R F Q N L G D M L A H
sp:O08749:DLDH_MOUSE	I G G P G G I E K N G G T C V G C S K G F G A T G S S I R F Q N L G D M L A H
tr:Q9VVL7:Q9VVL7_DROME	I G G P G G V E K E G G T C V G C S K G F G A T G S S I R Y E G L G D M L A H
sp:O17953:DLDH_CAEEL	I G G P G G V E K N G G T C V G C S K G F A A S G S S I R Y E G L G D M L A H
sp:P09624:DLDH_YEAST	I G G P A G V E K R G G T C V G C S K G N G A T G S S I R Y A G L G D M L A H

	whole protein		domain-based		contact-based	
protein	ident	simil	ident	simil	ident	simil
PDB:1jeh:chainA:P09624	1.0	1.0	1.0	1.0	1.0	1.0
PDB:1v59:chainA:P09624	1.0	1.0	1.0	1.0	1.0	1.0
sp:P09622:DLDH_HUMAN	0.53	0.81	0.57	0.85	0.84	0.88
sp:Q6P6R2:DLDH_RAT	0.53	0.82	0.57	0.85	0.84	0.88
sp:O08749:DLDH_MOUSE	0.53	0.82	0.57	0.85	0.84	0.88
tr:Q9VVL7:Q9VVL7_DROME	0.54	0.8	0.58	0.85	0.91	0.92
sp:O17953:DLDH_CAEEL	0.55	0.82	0.58	0.85	0.86	0.88
sp:P09624:DLDH_YEAST	1.0	1.0	1.0	1.0	1.0	1.0

CG7430 (UniProt:Q9VVL7) annotation
Cofactor: FAD Evidence=(RuleBase:RU003692); Note=Binds 1 FAD per subunit. (RuleBase:RU003692);
Miscellaneous: The active site is a redox-active disulfide bond. (RuleBase:RU003692).

(Information from UniProt)

dld-1 (WBGene00010794) associated phenotypes
lethal, sterile

(Information from WormBase)

dld-1 (UniProt:O17953) annotation
Cofactor: FADNote=Binds 1 FAD per subunit. ;
Subcellular location: Mitochondrion matrix
Miscellaneous: The active site is a redox-active disulfide bond.

(Information from UniProt)

NADPH–cytochrome P450 reductase

Best gene implication in ageing for this target family came from gene F1LQC7 via mapping the annotation from RGD 3186 annotated in RGD 2014-03-11. Annotation GO 7568 (aging) was Inferred from Expression Pattern

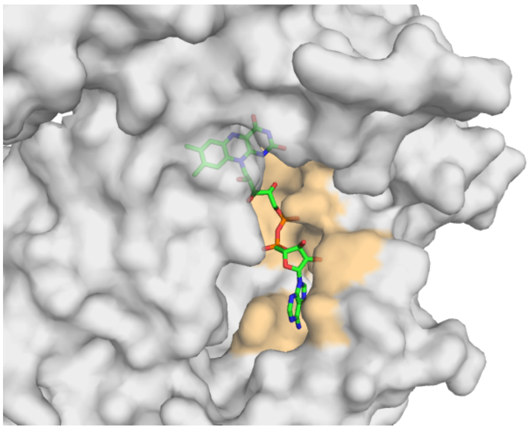

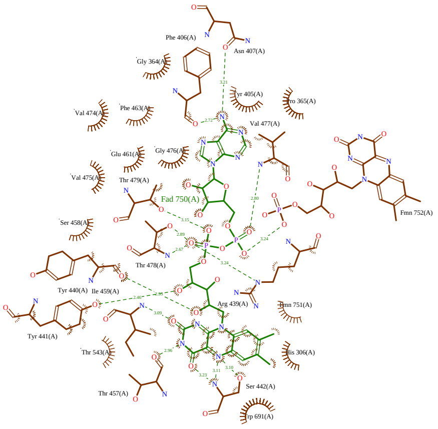

protein	amino acids contacts (binding site)
PDB:2bf4:chainA:P16603	H G P Y F N R Y Y S T S I E F V V G V T T T W
PDB:2bn4:chainA:P16603	H G P Y F N R Y Y S T S I E F V V G V T T T W
PDB:2bpo:chainA:P16603	H G P Y F N R Y Y S T S I E F V V G V T T T W
tr:E7ESA7:E7ESA7_HUMAN	H S P C P T R Y Y S T V A L Y H Y G V C S T F
sp:P35228:NOS2_HUMAN	H T P S P T R F Y S T V A V Y H H G V C S T F
sp:P29474:NOS3_HUMAN	H S P C P T R Y Y S T V A L Y H Y G V C S T F
tr:B3VK56:B3VK56_HUMAN	H T P N P T R Y Y S T V A V Y H H G V C S T F
sp:P29475:NOS1_HUMAN	H T P N P T R Y Y S T V A V Y H H G V C S T F
tr:F1LSH9:F1LSH9_RAT	H T P N P T R Y Y S T V A V Y H H G V C S T F
tr:F1LQC7:F1LQC7_RAT	H S P C P T R Y Y S T V A L Y H Y G V C S T F
tr:D3ZEM9:D3ZEM9_RAT	H T P N P T R Y Y S T V A V Y H H G V C S T F
tr:F1LQL1:F1LQL1_RAT	H T P N P T R Y Y S T V A V Y H H G V C S T F
tr:D3ZEW7:D3ZEW7_RAT	H T P N P T R Y Y S T V A V Y H H G V C S T F
sp:P29477:NOS2_MOUSE	H T P N P T R Y Y S T V A V Y H H G V C S T F
sp:P70313:NOS3_MOUSE	H S P C P T R Y Y S T I A L Y H Y G V C S T F
sp:Q9Z0J4:NOS1_MOUSE	H T P N P T R Y Y S T V A V Y H H G V C S T F
tr:S4R255:S4R255_MOUSE	H T P N P T R Y Y S T V A V Y H H G V C S T F
tr:F8WGF2:F8WGF2_MOUSE	H T P N P T R Y Y S T V A V Y H H G V C S T F
sp:Q27571:NOS_DROME	H T P L P H R F Y S T V A V Y R Y G V C S T F
sp:P16603:NCPR_YEAST	H G P Y F N R Y Y S T S I E F V V G V T T T W

protein	whole protein ident	simil	domain-based ident	simil	contact-based ident	simil
PDB:2bf4:chainA:P16603	1.0	1.0	1.0	1.0	1.0	1.0
PDB:2bn4:chainA:P16603	1.0	1.0	1.0	1.0	1.0	1.0
PDB:2bpo:chainA:P16603	1.0	1.0	1.0	1.0	1.0	1.0
tr:E7ESA7:E7ESA7_HUMAN	0.15	0.39	0.24	0.58	0.43	0.52
sp:P35228:NOS2_HUMAN	0.12	0.34	0.24	0.61	0.39	0.54
sp:P29474:NOS3_HUMAN	0.13	0.33	0.24	0.58	0.43	0.52
tr:B3VK56:B3VK56_HUMAN	0.11	0.31	0.24	0.59	0.43	0.54
sp:P29475:NOS1_HUMAN	0.11	0.31	0.24	0.59	0.43	0.54
tr:F1LSH9:F1LSH9_RAT	0.13	0.35	0.25	0.62	0.43	0.54
tr:F1LQC7:F1LQC7_RAT	0.12	0.33	0.23	0.57	0.43	0.52
tr:D3ZEM9:D3ZEM9_RAT	0.12	0.33	0.24	0.6	0.43	0.54
tr:F1LQL1:F1LQL1_RAT	0.11	0.31	0.24	0.6	0.43	0.54
tr:D3ZEW7:D3ZEW7_RAT	0.11	0.3	0.24	0.6	0.43	0.54
sp:P29477:NOS2_MOUSE	0.13	0.34	0.25	0.62	0.43	0.54
sp:P70313:NOS3_MOUSE	0.12	0.33	0.23	0.57	0.43	0.51
sp:Q9Z0J4:NOS1_MOUSE	0.11	0.31	0.24	0.6	0.43	0.54
tr:S4R255:S4R255_MOUSE	0.11	0.31	0.24	0.6	0.43	0.54
tr:F8WGF2:F8WGF2_MOUSE	0.11	0.3	0.24	0.6	0.43	0.54
sp:Q27571:NOS_DROME	0.11	0.31	0.24	0.6	0.39	0.59
sp:P16603:NCPR_YEAST	1.0	1.0	1.0	1.0	1.0	1.0

Nos (FBgn0011676) associated phenotypes

chemical resistant, developmental rate defective, dominant, eclosion defective, increased cell size, large body, neuroanatomy defective, partially lethal - majority die, small body, some die during pupal stage

(Information from FlyBase)

Nos (UniProt:Q27571) annotation

Function: Produces nitric oxide (NO) which is a messenger molecule with diverse functions throughout the body. Truncated isoforms (isoform 3-isoform 6) are able to form intracellular complexes with the full length protein and serve as dominant negative inhibitors of the enzyme activity. (PubMed:11526108, PubMed:12804606, PubMed:7568075).
Cofactor: heme
Cofactor: FADNote=Binds 1 FAD. ;
Cofactor: FMNNote=Binds 1 FMN. ;
Enzyme regulation: Stimulated by calcium/calmodulin. (PubMed:7568075).
Developmental stage: Isoform 3 is expressed in larvae only. Isoform 4, isoform 5, isoform 6 and isoform 10 are expressed throughout development from embryos to adults. (PubMed:11526108).

(Information from UniProt)

Xanthine dehydrogenase/oxidase

Best gene implication in ageing for this target family came from gene P10351 annotated in UniProt release 2014_02. Annotation GO 8340 (determination of adult lifespan) was Inferred from Mutant Phenotype

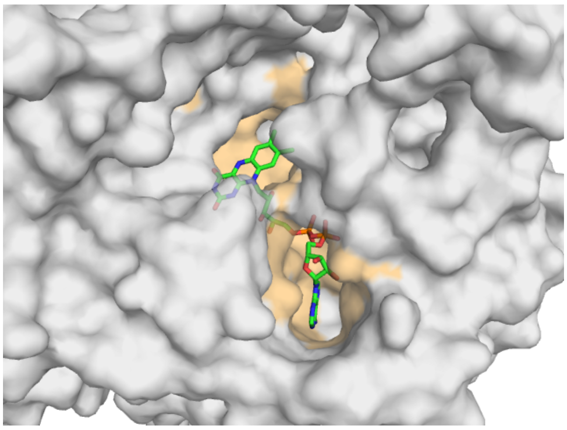 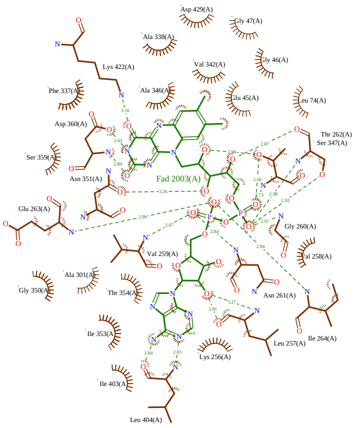

protein	amino acids contacts (binding site)
PDB:2ckj:chainA:P47989	E G G L K L V V G N T E I A F A V A S G N I T S D I L K - D
PDB:2e1q:chainA:P47989	E G G L K L V V G N T E I A F A V A S G N I T S D I L K D D
sp:P47989:XDH_HUMAN	E G G L K L V V G N T E I A F A V A S G N I T S D I L K D D
tr:F1LQS6:F1LQS6_RAT	E G G L K L V V G N T E I A F A V A S G N I T S D I L K D D
sp:Q00519:XDH_MOUSE	E G G L K L V V G N T E I A F A V A S G N I T S D I L K D D
sp:P10351:XDH_DROME	E G G L K L V V G N T E V A F A I A C G N M T S D V L K D D
tr:O17892:O17892_CAEEL	E G G L R L I S G N S E L T F A V A S G N A T S D I I K D D

protein	whole protein ident	whole protein simil	domain-based ident	domain-based simil	contact-based ident	contact-based simil
PDB:2ckj:chainA:P47989	1.0	1.0	1.0	1.0	0.97	0.93
PDB:2e1q:chainA:P47989	1.0	1.0	1.0	1.0	1.0	1.0
sp:P47989:XDH_HUMAN	1.0	1.0	1.0	1.0	1.0	1.0
tr:F1LQS6:F1LQS6_RAT	0.9	0.97	0.9	0.97	1.0	1.0
sp:Q00519:XDH_MOUSE	0.89	0.97	0.87	0.96	1.0	1.0
sp:P10351:XDH_DROME	0.52	0.83	0.49	0.83	0.83	0.94
tr:O17892:O17892_CAEEL	0.46	0.78	0.46	0.81	0.73	0.88

ry (FBgn0003308) associated phenotypes

chemical conditional, chemical sensitive, heat sensitive, partially, partially lethal, partially lethal - majority die, radiation sensitive, short lived

(Information from FlyBase)

ry (UniProt:P10351) annotation

Function: Key enzyme in purine degradation. Catalyzes the oxidation of hypoxanthine to xanthine. Catalyzes the oxidation of xanthine to uric acid (By similarity).
Cofactor: [2Fe-2S] cluster Evidence=; Note=Binds 2 [2Fe-2S] clusters. ;
Cofactor: FAD
Cofactor: Mo-molybdopterin Evidence=; Note=Binds 1 Mo-molybdopterin (Mo-MPT) cofactor per subunit. ;
Subunit: Homodimer.
Subcellular location: Peroxisome

(Information from UniProt)

Apoptosis-inducing factor 1, mitochondrial

Best gene implication in ageing for this target family came from gene O95831 via mapping the annotation from Ensembl ENSG00000156709 via mapping the annotation from EntrezGene 9131 via mapping the annotation from GenAgeHuman 0135 annotated in GenAge release 17.

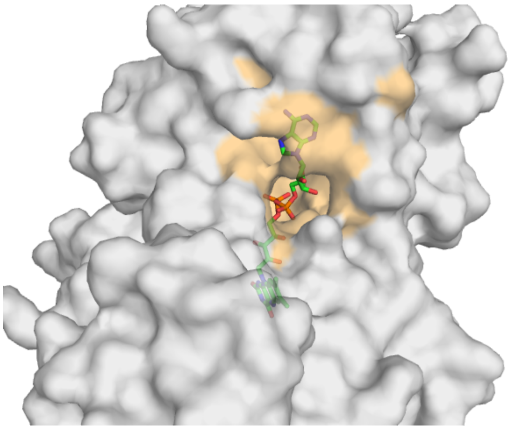
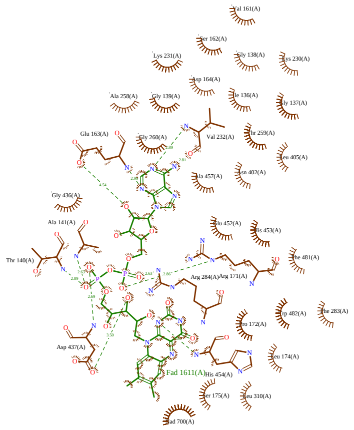

protein	amino acids contacts (binding site)
PDB:1gv4:chainA:Q9Z0X1	I G G G T A V S E D R P L S K K K V A T G G F R L N L G D E H H A F W
PDB:3gd3:chainA:Q9Z0X1	I G G G T A V S E D R P L S K K K V A T G G F R L N L G D E H H A F W
PDB:3gd4:chainA:Q9Z0X1	I G G G T A V S E D R P L S K K K V A T G G F R L N L G D E H H A F W
sp:O95831:AIFM1_HUMAN	I G G G T A V S E D R P L S K K K V A T G G F R L N L G D E H H A F W
sp:Q9JM53:AIFM1_RAT	I G G G T A V S E D R P L S K K K V A T G G F R L N L G D E H H A F W
tr:B1AU25:B1AU25_MOUSE	I G G G T A V S E D R P L S K K K V A T G G F R L N L G D E H H A F W
sp:Q9Z0X1:AIFM1_MOUSE	I G G G T A V S E D R P L S K K K V A T G G F R L N L G D E H H A F W
sp:Q9VQ79:AIFM1_DROME	I G G G T A I S N E R P L S K F S V A T G C Y R I N L G D E H H S F W
tr:E8NH83:E8NH83_DROME	I G G G T A I S N E R P L S K F S V A T G C Y R I N L G D E H H S F W
tr:K8ESD4:K8ESD4_CAEEL	I G S G T A I G E E R P L S K R K A A T G V F H L N I G A S S W A F F
tr:Q8HJ11:Q8HJ11_CAEEL	I G S G T A I G E E R P L S K R K A A T G V F H L N I G A S S W A F F
tr:Q9U229:Q9U229_CAEEL	I G S G T A I G E E R P L S K R K A A T G V F H L N I G A S S W A F F

	whole protein		domain-based		contact-based	
protein	ident	simil	ident	simil	ident	simil
PDB:1gv4:chainA:Q9Z0X1	1.0	1.0	1.0	1.0	1.0	1.0
PDB:3gd3:chainA:Q9Z0X1	1.0	1.0	1.0	1.0	1.0	1.0
PDB:3gd4:chainA:Q9Z0X1	1.0	1.0	1.0	1.0	1.0	1.0
sp:O95831:AIFM1_HUMAN	0.92	0.98	0.94	0.98	1.0	1.0
sp:Q9JM53:AIFM1_RAT	0.98	0.99	0.99	1.0	1.0	1.0
tr:B1AU25:B1AU25_MOUSE	0.95	0.97	1.0	1.0	1.0	1.0
sp:Q9Z0X1:AIFM1_MOUSE	1.0	1.0	1.0	1.0	1.0	1.0
sp:Q9VQ79:AIFM1_DROME	0.37	0.66	0.49	0.82	0.74	0.83
tr:E8NH83:E8NH83_DROME	0.35	0.64	0.49	0.82	0.74	0.83
tr:K8ESD4:K8ESD4_CAEEL	0.28	0.57	0.35	0.69	0.6	0.76
tr:Q8HJ11:Q8HJ11_CAEEL	0.26	0.57	0.35	0.7	0.6	0.76
tr:Q9U229:Q9U229_CAEEL	0.26	0.57	0.35	0.7	0.6	0.76

AIF (FBgn0031392) associated phenotypes

decreased cell death, dominant, immune response defective, increased cell death, partially, small body

(Information from FlyBase)

AIF (UniProt:Q9VQ79) annotation

Function: Probable NADH oxidoreductase (By similarity). Mitochondrial effector of cell death that plays roles in developmentally regulated cell death and normal mitochondrial function. (, PubMed:18309327).
Cofactor: FAD
Subcellular location: Mitochondrion intermembrane space (PubMed:18309327).
Developmental stage: Expressed both maternally and zygotically throughout development with slightly lower levels during pupation. (PubMed:18309327).
Disruption phenotype: Loss of zygotic expression results in decreased embryonic cell death and the persistence of differentiated neuronal cells along the ventral nerve cord at late embryonic stages. Embryos that do hatch undergo growth arrest at early larval stages, accompanied by mitochondrial

respiratory dysfunction. (PubMed:18309327).

(Information from UniProt)
